# The ATM and ATR inhibitors CGK733 and caffeine suppress cyclin D1 levels and inhibit cell proliferation

**DOI:** 10.1186/1748-717X-4-51

**Published:** 2009-11-10

**Authors:** John P Alao, Per Sunnerhagen

**Affiliations:** 1 Department of Cell and Molecular Biology, Lundberg Laboratory University of Gothenburg, P.O. Box 462, S-405 30 Göteborg, Sweden

## Abstract

The ataxia telangiectasia mutated (ATM) and the ATM- related (ATR) kinases play a central role in facilitating the resistance of cancer cells to genotoxic treatment regimens. The components of the ATM and ATR regulated signaling pathways thus provide attractive pharmacological targets, since their inhibition enhances cellular sensitivity to chemo- and radiotherapy. Caffeine as well as more specific inhibitors of ATM (KU55933) or ATM and ATR (CGK733) have recently been shown to induce cell death in drug-induced senescent tumor cells. Addition of these agents to cancer cells previously rendered senescent by exposure to genotoxins suppressed the ATM mediated p21 expression required for the survival of these cells. The precise molecular pharmacology of these agents however, is not well characterized. Herein, we report that caffeine, CGK733, and to a lesser extent KU55933, inhibit the proliferation of otherwise untreated human cancer and non-transformed mouse fibroblast cell lines. Exposure of human cancer cell lines to caffeine and CGK733 was associated with a rapid decline in cyclin D1 protein levels and a reduction in the levels of both phosphorylated and total retinoblastoma protein (RB). Our studies suggest that observations based on the effects of these compounds on cell proliferation and survival must be interpreted with caution. The differential effects of caffeine/CGK733 and KU55933 on cyclin D1 protein levels suggest that these agents will exhibit dissimilar molecular pharmacological profiles.

## Background

ATM and ATR cooperate to mediate cellular responses to DNA damage, following exposure to diverse genotoxic agents. These include induction of cell cycle arrest, DNA repair, maintenance of genomic stability, induction of premature senescence and cell death [1-3]. The coordinated activation of these processes has been defined as the DNA damage response pathway (DDR). Initial studies demonstrated that inhibition of ATM and ATR by caffeine significantly enhanced cellular sensitivity to ionizing radiation (IR) [4]. Inhibiting ATM, ATR or their downstream targets thus serves to widen the therapeutic window of genotoxic anti-cancer therapeutics by sensitizing cancer cells to these agents (reviewed [5]). The relative non-specificity of caffeine has lead to the search for more specific inhibitors of ATM and ATR. The small molecule inhibitor 2-morpholin-4-yl-6-thianthren-1-yl-pyran-4-one (KU55933) has been shown to specifically inhibit ATM in the low nanomolar range (IC_50_:12.9 nM). In contrast, KU55933 did not inhibit ATR at doses of up to 100 μM [6]. KU55933 has been shown to sensitize cancer cells to both IR and chemotherapeutic agents [6,7]. CGK733, a thiourea-containing compound, was originally identified as an inhibitor of ATM and ATR with an IC_50 _of ~200 nM towards both kinases [8]. This study was subsequently retracted, leaving the precise molecular pharmacology of this compound unclear. Additional studies suggest however, that CGK733 inhibits ATM and ATR [9-12]. More recently, caffeine, CGK733 and KU55933 have been shown to induce cell death in prematurely senescent breast cancer cells [13]. The induction of premature senescence by genotoxic agents contributes to drug sensitivity and is primarily (but not solely) dependent on p53-induced p21 expression [14,15]. Cancer cells that have undergone drug-induced premature senescence are less sensitive to pro-apoptotic signaling and can re-enter the cell cycle [16-20]. The study by Crescenzi *et al*. [13] suggests that ATM is required for the maintenance of the premature senescent phenotype and hence the survival of cancer cells exposed to genotoxins. Combining ATM and/or ATR inhibitors with genotoxins may thus further enhance the cytotoxicity of these agents, by preventing drug induced senescence as a therapeutic outcome [13]. The molecular pharmacology of inhibitors like CGK733 and KU55933 will require further characterization. ATR unlike ATM regulates cell cycle progression in the absence of DNA damage and is required for the viability of proliferating human and mouse cells [1]. Inhibitors that target both ATM and ATR are thus likely to exhibit pharmacological profiles that are distinct from ATM selective inhibitors. It is also likely, that the genetic make up of a particular subset of cancer cells influences their relative sensitivity to ATM and/or ATR inhibitors [21,22].

## Methods

### Reagents

Stock solutions of caffeine (100 mM in water) (Sigma Aldrich, Stockholm, Sweden), CGK733 (20 mM) and KU55933 (10 mM) (Calbiochem, VWR International AB, Stockholm, Sweden) dissolved in dimethyl sulphoxide (DMSO) were stored at -20°C. Lithium Chloride (40 mM) (Sigma-Aldrich) was dissolved in sterile distilled water and stored at 4°C. The proteasome inhibitor MG132 (50 mM) (Sigma- Aldrich) was dissolved in DMSO and stored at -20°C. Caspase inhibitor I (40 mM) (Z-VAD (OMe)-FMK (Calbiochem) was dissolved in DMSO and stored at -20°C. Monoclonal antibodies raised against cyclin D1 (DCS-6) (Santa Cruz Biotechnology, Santa Cruz, CA), RB (G3-245) (Becton Dickinson AB, Stockholm, Sweden), α- Tubulin (Sigma- Aldrich), and Hsp60 (Abcam, Cambridge, United Kingdom) were used.

### Cell culture

LNCaP, MCF-7, MDA-MB436 and T47D cells were cultured in RPMI 1640 supplemented with 10% (v/v) fetal calf serum, 2 mM L-glutamine, 100 units/ml penicillin and 100 μg/ml streptomycin at 37°C in humidified 5% CO_2_. HCT116 and BALB/c 3T3 cells were cultured in Dulbecco's modified eagle medium (DMEM) supplemented with 10% (v/v) fetal calf serum, 2 mM L-glutamine, 100 units/ml penicillin and 100 μg/ml streptomycin at 37°C in humidified 5% CO_2_.

### Cell proliferation assay

Cells were seeded in 96-well plates at a predetermined optimal cell density to ensure exponential growth for duration of the assay. After a 24 h preincubation, growth medium was replaced with experimental medium containing the appropriate drug concentrations or 0.1% (v/v) vehicle control. After a 48 h incubation, cell proliferation was estimated using the sulforhodamine B colorimetric assay [23] and expressed as the mean ± SE for six replicates as a percentage of vehicle control (taken as 100%). Experiments were performed independently at least three times. Statistical analyses were performed using a two-tailed Student's *t *test. P < 0.05 was considered to be statistically significant.

### Immunoblotting

Cells treated as indicated were harvested in 5 ml of medium, pelleted by centrifugation (1,600 rpm for 5 min at 4°C), washed twice with ice-cold phosphate buffered saline (PBS) and lysed in ice-cold HEPES buffer [50 mM HEPES (pH 7.5), 10 mM NaCl, 5 mM MgCl_2_, 1 mM EDTA, 10% (v/v) glycerol, 1% (v/v) Triton X-100 and a cocktail of protease inhibitors (Roche Diagnostics Scandinavia AB, Bromma, Sweden)] on ice for 30 min. Lysates were clarified by centrifugation (13,000 rpm for 15 min at 4°C) and the supernatants then either analyzed immediately or stored at -80°C. Equivalent amounts of protein (20 - 50 μg) from total cell lysates were resolved by SDS-PAGE and transferred onto 'nitrocellulose membranes. Membranes were blocked in blocking buffer [5% (w/v) nonfat dried milk, 150 mM NaCl, 10 mM Tris (pH 8.0) and 0.05% (v/v) Tween 20]. Proteins were detected by incubation with primary antibodies at appropriate dilutions in blocking buffer overnight at 4°C. Blots were then incubated at room temperature with horseradish peroxidase-conjugated secondary antibody. Bands were visualized by enhanced chemiluminescence (Supersignal West Pico; Pierce, Nordic Biolabs AB, Täby, Sweden) followed by exposure to autoradiography film (General Electric Bio-Sciences, Uppsala, Sweden).

### Immunofluorescence microscopy

MCF-7 cells were grown on sterile glass coverslips in 6- well plates to 80% confluence in media before being washed three times in PBS. Cells were fixed in 4% formaldehyde/PBS at room temperature for 10 minutes. Coverslips were washed twice in PBS and permeabilized in 0.2% Triton X100/PBS for 15 minutes. Following another three washes in PBS, coverslips were blocked in 3% bovine serum albumen (BSA)/PBS at room temperature for 30 min. Antibodies to Cyclin D1 (DCS-6) (Santa Cruz) (1:50 dilution) were applied in 3% BSA/PBS medium overnight. Cells were washed then washed 3 times in PBS, and incubated with a rhodamine (TRITC)- conjugated goat anti- mouse secondary antibody (1:200) (Jackson Immunoresearch, Fisher Scientific AB, Gothenburg, Sweden) at room temperature for 1 h. After a final 3 washes, coverslips were mounted on glass slides with Vectorshield containing 4', 6'-diamidino-2-phenylindole(DAPI) (Vector Laboratories Ltd., Peterborough, United Kingdom). Images were obtained with a Zeiss AxioCam on a Zeiss Axioplan 2 microscope with a 100 × objective using the appropriate filter sets.

## Results

**Figure 1:**
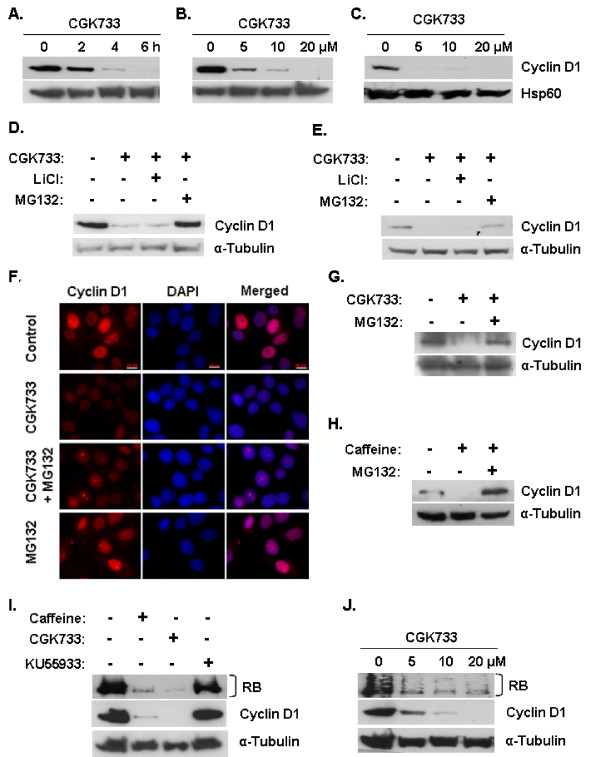
**Effect of CGK733, caffeine and KU55933 on cyclin D1 stability**. **A**. MCF-7 breast cancer cells were cultured with 10 μM CGK733 for the indicated times. Total lysates were resolved by SDS- PAGE and analyzed for cyclin D1 expression. Gel loading was monitored with an antibody raised against Hsp60. **B**. MCF-7 cells were cultured with the indicated concentrations of CGK733 for 6 h and analyzed as in A. **C**. T47D breast cancer cells were treated as in B. **D**. MCF-7 cells were incubated with 10 μM CGK733 alone or in the presence of 40 mM LiCl or 25 μM MG132 for 6 h and analyzed for cyclin D1 expression. Gel loading was monitored with an antibody directed against α- Tubulin. **E**. T47D cells were treated as in D. **F**. MCF-7 cells were grown on coverslips and treated with 10 μM CGK733 ± 25 μM MG132 for 6 h. Cyclin D1 expression was determined by indirect immunofluorescence microscopy as described in Materials and methods. Scale bar 10 μm. **G**. LnCap prostate cancer cells were treated and analyzed as in D. **H**. MCF-7 cells were cultured with 5 mM caffeine ± 25 μM MG132 for 6 h and analyzed as in D. **I**. MCF-7 cells were cultured in the presence of 5 mM caffeine, 10 μM CGK733 or 20 μM KU55933 for 24 h and analyzed for RB and cyclin D1 expression. **J**. MCF-7 cells were cultured with the indicated concentrations of CGK733 for 24 h and analyzed as in I.

In this work, we observe that CGK733 induces the loss of cyclin D1 via the ubiquitin- dependent proteasomal degradation pathway in MCF-7 and T47D breast cancer cell lines. Culture of MCF-7 breast cancer cells with 10 μM CGK733 induced a detectable decline of cyclin D1 levels within 2 h of exposure, and this effect was maximal between 4 and 6 h after exposure (Figure 1A). CGK733 induced the loss of cyclin D1 expression at concentrations as low as 5 μM and this activity was maximal at 10 to 20 μM (Figure 1B). CGK733 similarly induced loss of cyclin D1 protein in T47D cells at these concentrations (Figure 1C). The phosphorylation of cyclin D1 residue threonine 286 (T286) by GSK3β greatly enhances its degradation by the ubiquitin- 26S proteasome pathway. Several agents that induce cyclin D1 ablation have been shown to do so in a GSK3β dependent manner [24]. The CGK733 induced attenuation of cyclin D1 levels was inhibited by the 26S proteasome inhibitor MG132 but not the GSK3β inhibitor lithium chloride (LiCl) in MCF-7 and T47D cells (Figure 1D and 1E). CGK733 thus induces the loss of cyclin D1 protein via the ubiquitin- 26S proteasome pathway independently of GSK3β mediated T286 phosphorylation. GSK3β regulates cyclin D1 stability by facilitating its CRM1-dependent nuclear export and subsequent degradation within the cytoplasm [25,26]. Accordingly, CGK733 did not affect the subcellular localization of cyclin D1 (Figure 1F). CGK733 also induced ubiquitin-dependent loss of cyclin D1 in LNCaP prostate cancer cells (Figure 1G), indicating that this effect is not breast cancer specific. We also observed that caffeine induced the ubiquitin- dependent loss of cyclin D1 in MCF-7 cells (Figure 1H). In contrast, KU55933 did not affect cyclin D1 stability at the commonly used concentration of 10 μM for up to 24 h after exposure (Figure 1I). Cyclin D1 forms active kinase complexes with cyclin dependent kinase 4 (CDK4) or CDK6 that phosphorylate and hence inactivate the retinoblastoma tumor suppressor protein (RB) (reviewed in [27]). Caffeine and CGK733 but not KU55933 induced a significant decline in the levels of both phosphorylated and total RB protein levels in MCF-7 cells (Figure 1I and 1J).

**Figure 2:**
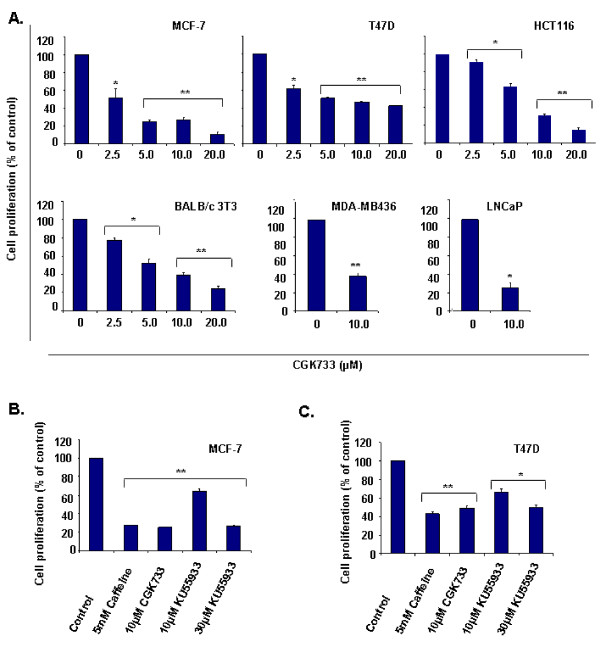
**Effect of CGK733, caffeine and KU55933 on cell proliferation**. **A**. MCF-7, T47D and MDA-MB436 breast cancer cells, LNCaP prostate cancer cells, HCT116 colon cancer cells and BALB/c 3T3 fibroblasts were cultured with the indicated doses of CGK733 for 48 h. Cell proliferation was measured as described in Materials and methods. Results represent the mean ± S.E. from three independent experiments. *, *P *< 0.05, compared with control; **, *P *< 0.005, compared with control. **B**. MCF-7 cells were cultured with the indicated doses of caffeine, CGK733 and KU55933 for 48 h. Cell proliferation was monitored as in A. Results represent the mean ± S.E. from three independent experiments. **, *P *< 0.005, compared with control. **C**. T47D cells were cultured with the indicated doses of caffeine, CGK733 and KU55933 for 48 h. Cell proliferation was monitored as in A. Results represent the mean ± S.E. from three independent experiments. *, *P *< 0.05, compared with control; **, *P *< 0.005, compared with control.

Cyclin D1 activity is required for G1- S phase progression and the regulation of its expression and/or stability is often deregulated in cancer cells (reviewed in [24]). Previously, we demonstrated that cyclin D1 is essential for the proliferation of MCF-7 cancer cells [28]. We thus investigated the effect of caffeine, CGK733 and KU55933 on the proliferation of a panel of human cancer cell lines derived from solid tumors. Various studies have used CGK733 at concentrations ranging from 0.6- 40 μM (Table 1). In our study, CGK733 inhibited proliferation of MCF-7 and T47D estrogen receptor (ER) positive breast cancer cells, MDA-MB436 ER negative breast cancer cells, LnCap prostate cancer cells and HCT116 colon cancer cells (Figure 2A). Furthermore, CGK733 also suppressed proliferation of non- transformed mouse BALB/c 3T3 embryonic fibroblast cells (Figure 2A). The CGK733-mediated inhibition of proliferation was dose dependent and significant at doses as low as 2.5 μM. Culture of MCF-7 and T47D cells with 5 mM caffeine inhibited cell proliferation to a similar degree as CGK733 (Figure 2B). KU55933 maximally inhibits cellular ATM kinase activity at 10 μM and was originally reported not to inhibit cell proliferation at this concentration [6]. KU55933 has been used at doses ranging from 1 to 40 μM (Table 1), and later studies suggested that some cell lines exhibit sensitivity to this inhibitor at concentrations between 10 and 20 μM [21]. We observed that KU55933 inhibited proliferation of MCF-7 and T47D cells at concentrations ranging from 10 to 30 μM (Figure 2B and 2C). The ability of caffeine and CGK733 to induce the loss of cyclin D1 expression is thus likely to enhance their anti-proliferative activity at high concentrations.

**Table 1:** Inhibitors of ATM and/or ATR

**Study**	**Inhibitor/Concentration**			**Reference**
	**Caffeine**	**CGK733**	**KU55933**	
Hickson et al., 2004	**-**	**-**	0.03- 10 μM	[6]
Cowell et al., 2005	-	-	1-20 μM	[7]
Byrant and Helleday, 2006	-	-	2-20 μM	[21]
Nakai-Murakami et al., 2007	-	-	1 mM	[32]
Al-Minawi et al., 2008	2 mM	10 μM	-	[33]
Yamauchi et al., 2008	-	-	10 μM	[34]
Cruet-Hennequart et al., 2008	-	10 μM	10 μM	[10]
Goldstein et al., 2008	-	0.6 μM	-	[11]
Crescenzi et al., 2008	1-5 mM	10- 20 μM	20- 40 μM	[13]
Bhattacharya et al., 2008	-	20 μM	-	[9]

Selected studies involving the exposure of mammalian cell lines to the ATM and/or ATR inhibitors caffeine, CGK733 and KU55933.

**Figure 3:**
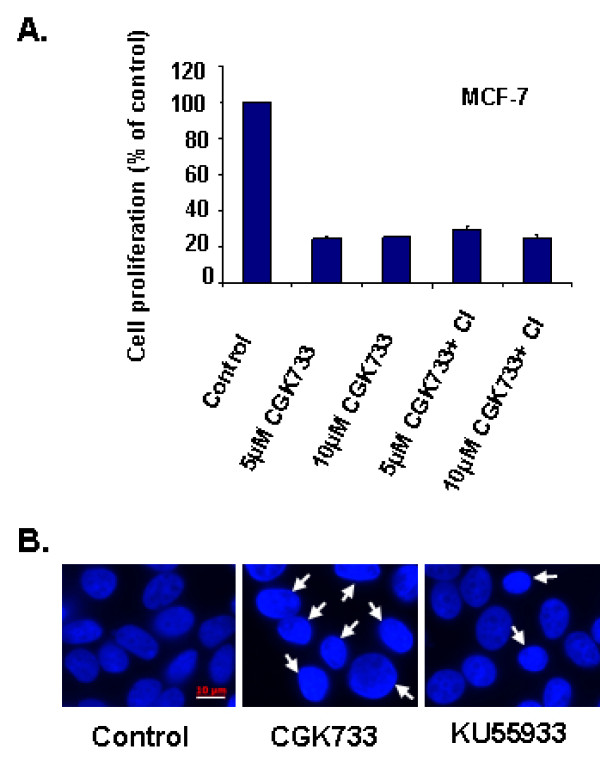
**Caspase independent inhibition of CGK733 on MCF-7 proliferation**. **A**. Cells were cultured with the indicated doses of CGK733 ± pan- caspase inhibitor z-VAD-fmk (40 μM) for 48 h. Cell proliferation was measured as described in Materials and methods. Results represent the mean ± S.E. from three independent experiments. **B**. MCF-7 cells were grown on coverslips and treated with 10 μM CGK733 or 10 μM KU55933 for 48 h. Cells were fixed and stained with DAPI as described in Materials and methods. White arrows denote condensed nuclei. Scale bar 10 μm.

Crescenzi *et al *[13] recently reported that caffeine (1- 5 mM), CGK733 (10 - 20 μM) and KU55933 (20 - 40 μM) induced senescent cancer cells to undergo caspase- dependent apoptosis. Our findings demonstrate, however, that these compounds inhibit the proliferation of otherwise untreated cancer and non- transformed cell lines (Figure 2). In contrast to that study however, pan-caspase inhibition did not suppress the anti-proliferative effect of CGK733 on MCF-7 cells in our experiments (Figure 3A). Although the nuclei of CGK733 treated cells appeared condensed, we did not detect the nuclear fragmentation normally detected in apoptotic MCF-7 cells (Figure 3B) [13]. Caffeine, CGK733 and KU55933 may thus suppress proliferation of senescent and non- senescent cancer cells via different mechanisms.

## Discussion

Small molecule selective inhibitors of ATM and/or ATR provide powerful tools for studies on the cellular functions of these kinases. Furthermore, these molecules may eventually be used alongside regular anti-cancer agents as chemo- and radiosensitizers (reviewed in [5]). In this study, we have observed that in contrast to the ATM selective inhibitor KU55933, ATM/ATR dual inhibitors such as caffeine and CGK733 can suppress cyclin D1 levels in cancer cell lines. A recent study suggests that mitogen-activated cyclin D1 expression is required for the induction of premature senescence [29]. Interestingly, cyclin D1 expression is elevated in cells that have been induced to undergo premature senescence [30]. The function of cyclin D1 in this non-dividing cell population has not been determined. Our findings show that single and dual ATM/ATR kinase inhibitors are not interchangeable and may thus differentially influence cell fate in a cell type and context dependent manner. KU55933 has been shown to enhance the radiosensitivity of cancer cells [6] but the impact of senescence suppression on this sensitizing effect remains unclear. Future studies will address how the effect(s) of caffeine and CGK733 on cyclin D1 expression, in turn impact on their chemo- and radiosensitizing properties. It remains unclear if the decline in cyclin D1 levels results from ATR inhibition, the inhibition of both ATM and ATR, or from the inhibition of an unknown target. It should be noted however, that the siRNA-mediated knockdown of ATR induced cyclin D1 accumulation in NIH 3T3 cells [31]. It is also conceivable, that the antiproliferative effects of ATM/ATR inhibitors observed at high does may result from off- target effects. It remains to be determined, if these agents exert cytotoxic effects on senescent cancer cells at lower doses [13]. Observations on cancer cell proliferation and survival based on the use of ATM/ATR inhibitors should thus be interpreted with caution.

## Competing interests

The authors declare that they have no competing interests.

## Authors' contributions

JPA and PS conceived and designed the study. JPA performed the experiments. JPA and PS analyzed and interpreted the data. JPA and PS drafted and wrote the manuscript.
